# Evisceration without Instruments

**Published:** 1878-11-20

**Authors:** J. T. Suggs

**Affiliations:** Texas


### EVISCERATION WITHOUT INSTRUMENTS.

BY. J. T. SUGGS, M. D., OF TEXAS.

I was called to a case of difficult labor. Mrs. A's second confinement had been in labor 36 hours, just seen by Dr. D. Dr. D. had Dr. A. called, after the case had been on hand 12 hours. Dr. A. found a shoulder presentation—the child dead. After getting the patient well under the influence of chloroform, I took the patient in hand. After repeated examinations, during several hours, I was unable to find either head or feet.

The Caesarian operation was proposed and insisted upon. I objected, because I did not think it proper to imperil the life of the mother, knowing that the child was dead.

After searching in vain amongst the instruments at hand, for an instrument with which to perform evisceration of the child, I seized the protruding arm, twisted it off at the shoulder, then with finger, cautiously tore the integuments and muscles, eviscerating in that way.

In a few minutes the delivery was completed. The child was large and well formed, with one exception; there were no bones in the head or face; the head felt like a small tumor, for which I had mistaken it, in making the examinations. The trouble was now explained; the head could not be recognized, on account of the deformity, and the child became immovably impacted. There is a great deal said in obstetrical works, about the danger of injuring the mother, in eviscerating the child. This case had a good recovery. A small amount of ingenuity, with a determined will, sometimes supplies the place of instruments. See case of occluded *os uteri* in New Orleans Medical and Surgical Journal, March, 1860.

The case above described, is such as I have never seen or heard of before, and I

submit it as a case showing the practicability of evisceration without instruments.

